# Supplementary material for: Inner core static tilt inferred from intradecadal oscillation in the Earth’s rotation
Source: Nat Commun. 2023 Dec 8;14:8130. doi: 10.1038/s41467-023-43894-9 (PMC10709600; doi:10.1038/s41467-023-43894-9)
Supplement: Supplementary file 1 — Supplementary Information [file 41467_2023_43894_MOESM1_ESM.pdf]

Supplementary Information for:

Inner core static tilt inferred from intradecadal oscillation in the Earth's rotation

Yachong An[1,3], Hao Ding[1,3]*, Zhifeng Chen[1], Wenbin Shen[1], Weiping Jiang[2]*

1 School of Geodesy and Geomatics, Hubei LuoJia Laboratory, Wuhan University, 430079, Wuhan, China

2 GNSS Research Center, Wuhan University, 430079, Wuhan, China

3 These authors contributed equally: Yachong An, Hao Ding

* Corresponding author. Email: dhaosgg@sgg.whu.edu.cn; wpjiang@whu.edu.cn

This file includes:

Supplementary Text

Supplementary Figure 1 to Figure 3

Supplementary References (1 and 2)

The execution of the stabilized AR-z spectrum

The Lorentzien power spectrum, Eq (9) in the main text, is constructed by "blindly" forcing the AR solution for each and every elementary Fourier frequency bin. An AR-z (Lorentzien) peak will appear in the frequency spectrum whenever a pole exists, signifying a harmonic signal at the frequency in question[1]. In the Fourier frequency bins (or the z-domain bins), where no actual signal exists, the apparent AR spectrum is due solely to noise in a statistically random way; a test code can be found in Ref. 1. To further suppress the error estimation caused by noise, i.e., constructing the stabilized AR-z spectrum[1], the Monte Carlo method is introduced. Adding an artificial white noise series to a data record will obtain an AR-z spectrum; this contains artificial white noise. This process is repeated K times ($K \geq 300$); the stabilized AR-z spectrum is finally obtained by raising the product spectrum to the K-th power. The end result is an accentuation of real-signal peaks against the reduced-variance background noise spectrum (see details in Ref. 1).

There are two important paraments for constructing the stabilized AR-z spectrum, the added noise level R and the Q factor for the target signal. For R, given that we used the MATLAB code 'randn.m' to produce the white noise which contains random 71 numbers (the used ΔLOD/PM have a 71yr long, 1949-2020), we selected R= 0.05 for the ΔLOD, and R= 6 for the PM. As for Q, since we did not know the Q of the target signal in advance, we chose a value close to infinity, $Q = 1\times10^{20}$.

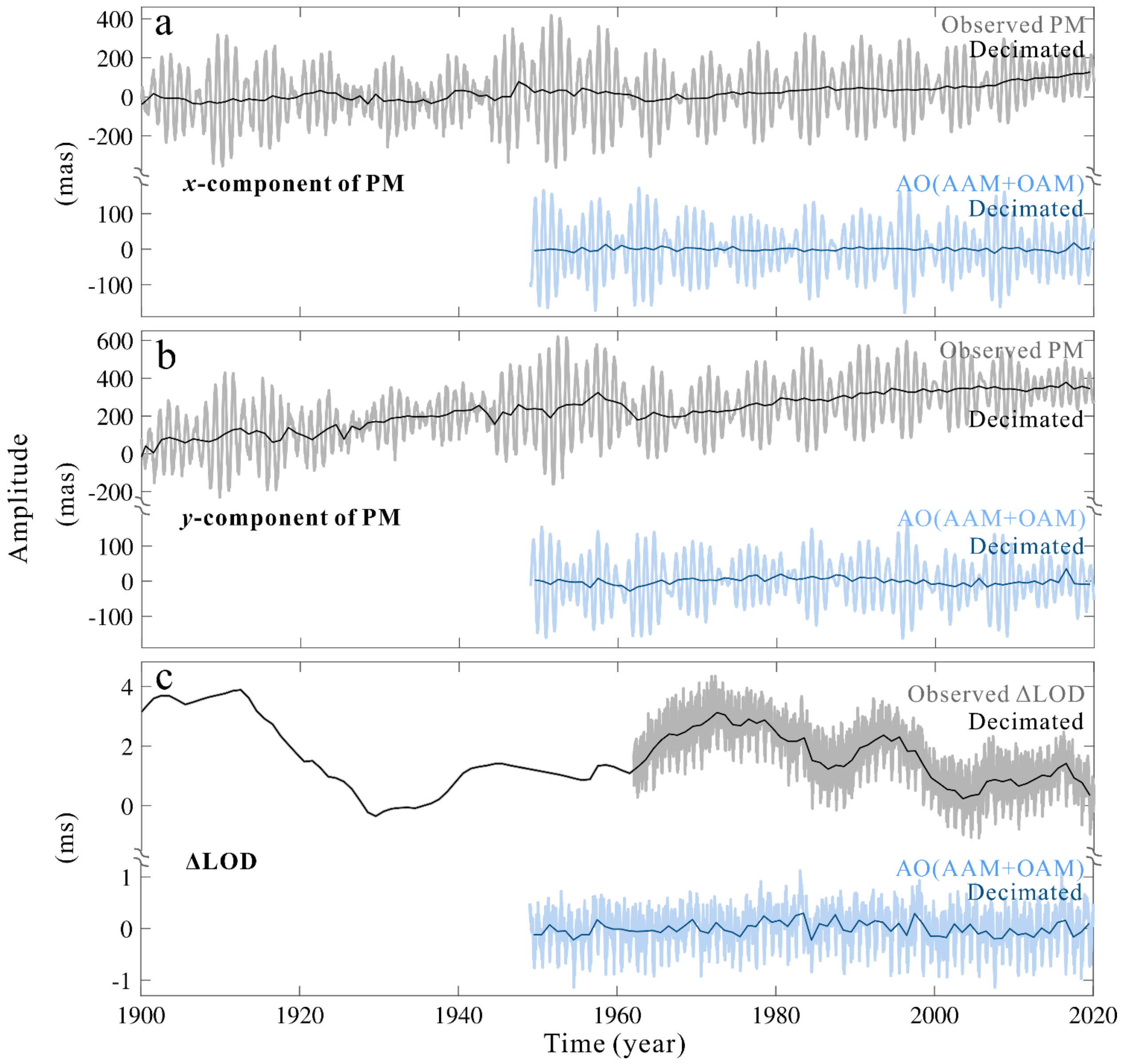

Supplementary Figure 1. (a) The *x* component of the polar motion (PM), (b) *y* component of the PM, and (c) length-of-day variation (ΔLOD) observation with their corresponding excited results by the atmospheric and oceanic angular momentum (AAM+OAM; AO). Here, the *x* and *y* components of the PM are the reported location of the celestial intermediate pole within the terrestrial reference frame, with the *x* component along the Greenwich meridian and the *y* component along 90°W longitude.

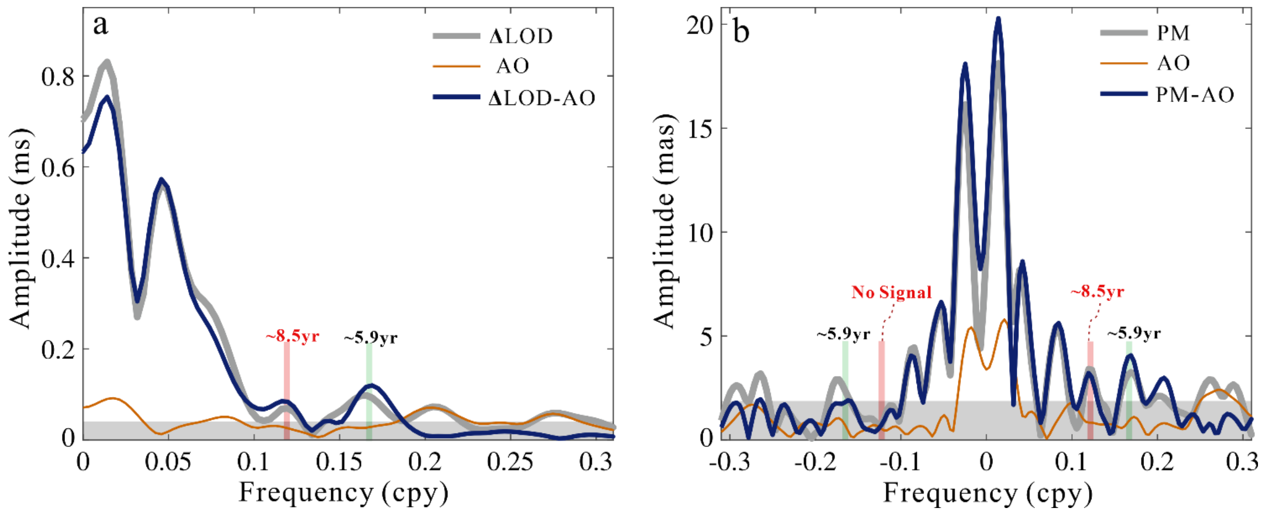

Supplementary Figure 2. (a) Fourier amplitude spectra of the observed length-of-day variation (ΔLOD) sequence (949.5-2019.5), the ΔLOD excited by atmospheric and oceanic angular momentum (AAM+OAM, abbreviated as AO), and the residual sequence (ΔLOD−AO). Panel (b) is similar to panel (a) but considers the polar motion (PM; $x-iy$). The grey shading represents the background noise level. Different from the AR-z spectra, in the target frequency band, only the ~5.9yr and the ~8.5yr signals can be identified in the Fourier spectra. The AO effects clearly have no significant contribution to the target frequency band. Using this figure, we find that only the ~8.5yr signal satisfies the special feature of the inner core wobble (ICW) of only having a positive frequency.

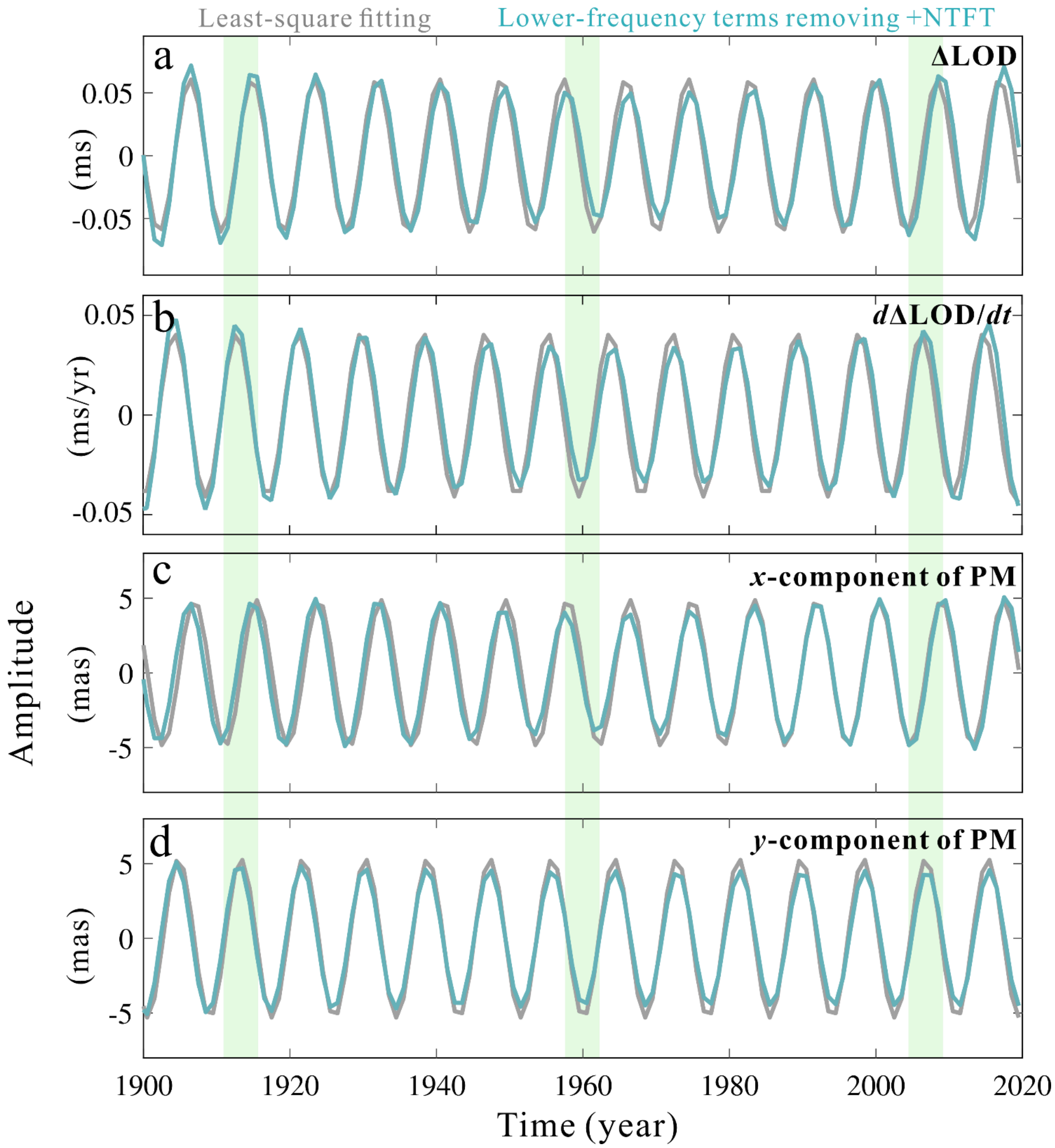

Supplementary Figure 3. The extracted ~8.5yr oscillations using the least-square fitting method (grey curves) and the normal time-frequency transform (NTFT)[2] combined with removing the low-frequency terms (cyan curves) from the (a) length-of-day variation (ΔLOD), (b) *d*ΔLOD/*dt*, (c) *x* component of the PM, and (d) *y*-component of the polar motion (PM). The results of the NTFT can be used to reconstruct the time-varying amplitude of the ~8.5yr oscillations and provide further validation of the results in the Main.

Supplementary References

1. Ding, H., & Chao, B. F. (2018). Application of stabilized AR-z spectrum in harmonic analysis for geophysics. *Journal of Geophysical Research: Solid Earth*, *123*(9), 8249-8259.
2. Liu, L., Hsu, H., & Grafarend, E. W. (2007). Normal Morlet wavelet transform and its application to the Earth's polar motion. *Journal of Geophysical Research: Solid Earth*, *112*(B8).
